# Poor-Law Infirmaries as Training Schools

**Published:** 1913-09-27

**Authors:** 


### POOR-LAW INFIRMARIES AS TRAINING SCHOOLS.

### A Consultative Staff for Poor-Law Institutions.

In Poor-Law institutional practice the medical superintendent has to play the part of specialist in all branches of medicine and surgery. It is obvious that cases must occasionally be met with where a consultant's opinion is advisable. It would be surprising if in the varied clinical material provided by Poor-Law patients such cases were rarities. At present no machinery exists under the Poor Law for such occasions. The ideal system would provide means whereby the pauper or destitute may, if his case demands it, secure the most expert opinion procurable. Nowadays such cases are forwarded to the general hospitals with a personal note from the medical superintendent explaining the points of the case. This is tantamount to a confession of the need for further advice. It is not a reflection on the medical superintendent's skill; rather the reverse, as the incident proves that he realises his limitations, and is not always willing to pose as an expert in all the intricate branches of medical art. It points also to a defect in Poor-Law medical administration. The Guardians (and they are responsible for cases sent from their institutions), as representing the ratepayers, have no right to sponge on institutions provided by a charitable public for other classes of patients.

In such circumstances there are only two alternatives. One is to seek the advice of a private consultant and to pay handsomely for the attention or advice. The other course is the appointment of a consultative staff for Poor-Law purposes, with allotted wards in a special institution. Many considerations can be urged in favour of the latter scheme. It is too much to ask that a consultative staff be appointed immediately to each Metropolitan infirmary. That reform will come in time.

The Metropolitan Guardians should allow the Metropolitan Asylums Board to provide an institution solely for Poor-Law consultative work. Experts and specialists, with the assistance of a resident staff, would be appointed to attend to the cases on which advice or consultation is sought or for treatment if permanently transferred. The Metropolitan Asylums Board already provide the Carshalton Hospital for tubercular cases in children and the Downs School for ringworm, each a specialised institution in a way. But this scheme is the natural corollary of such institutions. The Metropolitan Asylums Board have recently appointed a new dermatologist. Instead of his services being retained at a honorarium of £350 per annum solely for the purpose of "clinching" the diagnosis in a few doubtful cases of ringworm (cases where a bacteriological examination of the hairs would be more useful), wards could be delegated for dermatological cases under his supervision. Such cases would be those on which a consultant's opinion is considered necessary by some Poor-Law superintendent. Wards in the Park Hospital, where the ringworm cases are now housed, could be allotted for such work. Similarly, wards could be set aside for surgical or medical cases requiring consultation; a staff could be appointed solely for this work. The cases can be brought to this special institution either only for consultation or for the treatment advised by the consultant to be carried out in the wards allotted to him.

When this partial scheme has proved a success and paved the way to more ambitious ventures on these lines, other specialties may be considered.

In this way the Poor Law will have retained for consultative work the services of well-known consultants, men near or at the height of the profession in their particular specialty. A start would be made to place the Poor Law on an ideal medical basis. Poor-Law patients, especially the interesting difficult cases, would benefit; medical superintendents would have the skill of experts at their command, and a great reform would be quietly initiated.

For the success of this scheme, the co-operation of the Poor-Law medical superintendents in the Metropolitan area is an absolute necessity. Too frequently there is a tendency to enumerate all the difficulties in the way of carrying out such a scheme, while, granted good administration and hearty co-operation, no mention is made of the advantages of initiating such a reform. Difficulties are there only to be overcome. The time is mature; the institution, modern and fully equipped, is ready; the consultants are to be had; success depends on the scattered administrative unit heads who, by combination and a whole-hearted willingness, hold the key of the situation in their hands. Will the door be opened? H. C.

